# The Jidong Eye Cohort Study: objectives, design, and baseline characteristics

**DOI:** 10.1186/s40662-020-00223-1

**Published:** 2020-12-29

**Authors:** Kai Yang, Lele Cui, Guoyun Zhang, Xianwei Wang, Xiaoxuan Zhu, Yunfan Xiao, Binbin Su, Daiyu Song, Xinyao Zhang, Yang Zheng, Fan Lu, Jia Qu, Ming Li

**Affiliations:** 1 Eye Hospital and School of Ophthalmology and Optometry, Wenzhou Medical University, Wenzhou, Zhejiang China; 2 National Clinical Research Center for Ocular Diseases, Wenzhou, Zhejiang China; 3 Dezhou Center for Disease Control and Prevention, Dezhou, Shandong China

**Keywords:** Cohort, Risk factor, Visual impairment, Cardiovascular disease, Neurological disease, Epidemiology

## Abstract

**Background:**

To describe the objective, design and baseline characteristics of the Jidong Eye Cohort Study (JECS), a community-based cohort in China based on etiology, imaging and biomarkers. The JECS will clarify the pathogenesis of visual impairment and status of ocular indicators in the occurrence and progression of cardio-cerebrovascular and neurological diseases.

**Methods:**

Between August 2019 and January 2020, the JECS recruited consecutive participants aged 18 years and older from the Jidong communities in China. The demographic and clinical characteristics were collected by trained site personnel via face-to-face interviews. The relevant biological samples were also collected. The participants underwent comprehensive ophthalmic examination, such as retinal photography and optical coherence tomography (OCT) angiography. The following outcomes were measured annually: ocular vascular abnormality, optic nerve degeneration, cardiovascular diseases (CVD) and neurological diseases. The study will be performed until 2024.

**Results:**

Among 3377 participants, the average age was 45.0 ± 12.5 years and 1809 (53.6%) were women. Hypertension occurred in 825 individuals (25.0%), diabetes in 258 (7.7%), hyperglycemia in 474 (14.2%), and a CVD history in 100 (3.0%). The mean best-corrected visual acuity was 0.1 logMAR in the recruited subjects. The average OCT signal index was 8.2 ± 1.2. Additionally, the mean vessel densities for the entire measured area were 46.4% and 50.8% for the superficial and deep vascular complex, respectively. Mean area and perimeter of foveal avascular zone was 0.3 mm^2^ and 2.3 mm.

**Conclusions:**

The JECS is a large community-based prospective cohort in North China. Rich data collected from this study will provide the opportunity to identify risk factors, imaging, and biomarkers of visual impairment (either ocular vascular anomalies or optic nerve degeneration) and to evaluate their associations with CVD and neurological diseases.

## Background

Approximately 217 million individuals around the world had moderate to severe visual impairment in 2015, among whom 36 million are blind and approximately 80% reside in developing countries [1]. About 82% of individuals with blindness and 65% with mild to severe vision loss are aged 50 years or older [2]. Retinal and choroidal vascular abnormalities, such as diabetic retinopathy, retinal vein occlusion and age-related macular degeneration, are the most common causes of irreversible visual impairment. Optic nerve degeneration is also regarded as a cause of visual impairment [2–8]. Therefore, it is critical to identify risk factors of visual impairment to prevent occurrence of ocular vascular abnormalities and optic nerve degeneration among middle-aged or older individuals with the use of images and biomarkers [9–11].

Patients with ocular vascular abnormalities often develop cardiovascular diseases (CVD) due to the comorbidities of diabetes, hypertension and hyperlipidemia. According to the Global Burden of Disease Study, ischemic cardiovascular disease, including stroke, is the major leading cause of death [12, 13]. Additionally, cognitive impairment is a common issue worldwide with an estimated 75.6 million individuals developing dementia by 2030 and approximately $604 billion in social costs per year [14, 15]. Thus, identifying novel risk factors for vascular abnormalities and nerve degeneration is critical to improve the screening of CVD and cognitive impairment in the early stages. The pathological change in the cerebral vasculature could be reflected in the retinal blood vessel due to their similar anatomy and physiology [16]. Given that retinal blood vessels are more easily and directly assessed, ocular vascular abnormality is being considered a prediction tool for the development of cerebrovascular disease [16–20].

Taken together, this study aims to evaluate the pathogenesis of visual impairment, including retinal vascular abnormality and optic nerve degeneration, and explore its association with cardiovascular or neurological disease in a community-based population. In this report, we describe the objective, study design, baseline characteristics, and strengths and potential limitations of the Jidong Eye Cohort Study (JECS).

## Methods 

### Study design

The Jidong Eye Cohort Study (JECS) is a community-based, prospective, long-term follow-up observational study conducted by the Affiliated Eye Hospital of Wenzhou Medical University. The JECS was designed to identify the etiology, imaging and biomarkers of visual impairment and its subtypes, evaluate the associations of ophthalmic lesions with the risks of cardiovascular and neurological diseases, and facilitate the identification and evaluation of vascular abnormalities and nerve degeneration in the early phase.

**Fig. 1:**
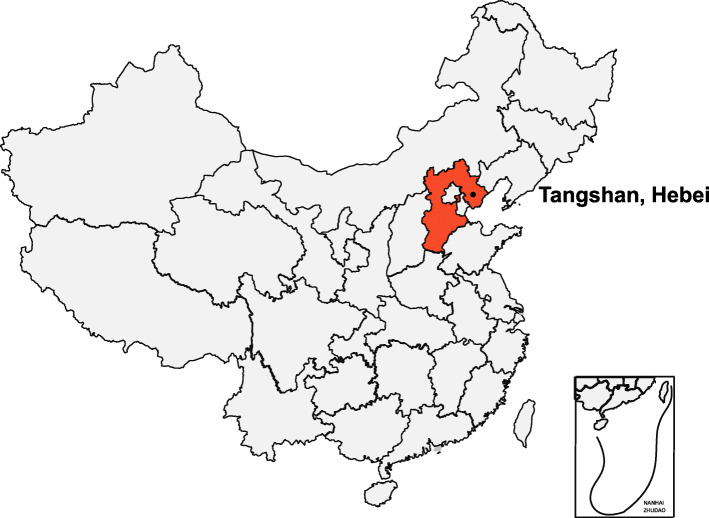
The geographical location of participants in JECS

The study was performed in the Jidong community of Tangshan city in Hebei province. The location of the study is illustrated in Fig. 1. The study complied with the guidelines of the Declaration of Helsinki and was approved by the Ethics Committee of the Staff Hospital of the Jidong Oil-Field of Chinese National Petroleum (China National Petroleum Corporation Jidong Oil-Field Branch Staff Hospital approval document of the medical ethics committee, 2018 YILUNZI 1). All participants signed an informed consent form.

### Specific aims

The primary objectives of this study were to clarify the pathogenesis of visual impairment from long-term follow-up observation in the Jidong community based on detailed etiological investigation, imaging and biomarkers, and establish a predictive model of visual impairment. The secondary objective of the JECS was to assess the association of CVD, including cerebrovascular disease, neurological disease, and other relevant disorders such as glucose metabolism and renal function, with ophthalmic disease.

### Participant enrollment

The study consecutively recruited participants who had a physical examination in the Staff Hospital of Jidong Oil-Field of China National Petroleum Corporation (the employees and retirees of the Jidong Oil-Field of Chinese National Petroleum Company or their relatives) from the Jidong community who were age 18 years and older and had the ability to provide informed consent.

Participants with missing blood samples or an uncompleted questionnaire were excluded in this report. Additionally, we exclude participants who had CVD, neurological disease, a vision acuity lower than 0.1 logMAR and eye disease, or whose images, including those from fundus photography and OCT angiography, were of poor quality in the subsequent follow-up.

### Baseline data collection

Baseline data were collected by the local clinicians and study personnel who were trained according to a standard protocol developed by the steering committee. The research investigators were trained by the field general office based on a standard protocol until they had the required capabilities. Research investigators enrolled consecutive participants, obtained informed consent and collected data by direct interviews.

A wide-ranging questionnaire comprised items such as demographics and lifestyle medical history. The demographics data included age, sex, marital status, occupation, income and education. The lifestyle data included items related to smoking, drinking, diet, exercise and the duration of nocturnal sleep. The medical history and current medication were also recorded. Women’s health questions were asked of female participants. Mental state was assessed by the Mini Mental Status Examination Scale (MMSE) and Montreal Cognitive Assessment (MoCA) [21–23]. The participants’ height and weight were measured using a digital scale (in kilograms). The body mass index (BMI) was calculated by dividing the weight by the height squared. The waist-hip circumference was measured using a wall-mounted tape (in centimeters). Blood pressure and pulse were measured using a digital automatic blood pressure monitor after resting for five minutes. The blood pressure of the individual was then taken as the mean between the two closest readings. The blood pressure was estimated according to the American College of Cardiology/American Heart Association (ACC/AHA) Guideline [24].

An electronic data system combined with the paper-based questionnaire was developed for data collection. Trained research investigators stored the data on a laptop and then uploaded the data with their electronic signature later. All imaging examination and laboratory test results were uploaded to the electronic data system as pictures directly. All the data elements were checked manually by several independent study personnel in the field general office throughout the study period. We randomly checked the completed data and regularly monitored centrally to ensure the high quality of data collection. Additionally, an independent contract research organization will perform quality control of all the monitored items. All data elements collected were deidentified before analysis.

### Biological sample collection

Food and drink were forbidden for 8 h before blood collection, and fasting elbow venous blood was collected by trained local clinicians in the morning and stored in vacuum tubes containing ethylenediaminetetraacetic acid and coagulation tubes. Hematological tests, such as fasting blood glucose, hemoglobin A1c (HBA1c), total cholesterol (TC), total triglycerides (TG), high-density lipoprotein (HDL-C), low-density lipoprotein (LDL-C), alanine aminotransferase, serum creatinine (Scr), and uric acid (UA) tests, were conducted first on fresh samples at the Central Laboratory of Jidong Oil-Field Hospital using an autoanalyzer (Hitachi, Tokyo, Japan). Additionally, blood samples were processed and the extracted serum, plasma and white blood cells were stored in the laboratory. Other biological samples, such as tongue coating samples, were also obtained. All the biological samples were stored in a -80 °C freezer.

### Ophthalmic examinations

The distance visual acuity (VA) of each eye (including uncorrected and corrected visual acuity) was measured using the Standard logarithmic VA Charts at a distance of 5 m. If line 0.1 was not identified at 1 m, the VA would be assessed by finger counting, hand movements, light perception, or no light perception along with the examination distance. We checked the right eye first followed by the left eye. Additionally, the refraction (including the diopter of sphere, diopter of column and axis) of the participant was measured using an autorefractor (KR800; Topcon Tokyo, Japan) to assess the participant’s refractive status. The central corneal thickness (CCT), aqueous depth (AD), lens thickness (LT), corneal curvature, pupil diameter and axial length (AL) were measured using optical low-coherence reflectometry (Lenstar 900 Optical Biometer; Haag-Streit, Koeniz, Switzerland). All participants were subjected to examinations of the anterior segments of the eye using a slit-lamp microscope, the peripheral anterior chamber depth using the Van Herick method, and cataract using the Lens Opacities Classification System (LOCS III). The posterior segments were also examined using a slit-lamp microscope with a 90-diopter lens and mainly examined the optic disc, retinal vessels and macula. All participants had also undergone digital fundus photography (CR2AF; Canon Tokyo, Japan).

RTVue XR-100 with a central wavelength of 840 nm and a bandwidth of 45 nm (RTVue XR Avanti with AngioVue; Optovue Fremont, CA, USA) were used to measure retinal complex perfusion (RCP) and retinal thickness. The levels of the retinal superficial (the vessel between the internal limiting membrane and the inner plexiform layer) and deep (the vessel between the inner plexiform layer and the outer plexiform layer) vascular complex and choriocapillaris blood flow and flow void were measured using the optical coherence tomography angiography (OCTA) mode. An image quality score ≥ 6 is considered qualified, otherwise it will be excluded. We analyzed the percent of blood flow and flow void in a certain area, which reflects the level of the blood perfusion in this sector. Additionally, choroidal thickness was measured using the Enhanced HD Line mode.

### Imaging data collection

All participants were recommended for the following protocols: cardio-cerebrovascular examination: brain CT, chest CT, resting 12-lead electrocardiogram (ECG) and rhythm strip recorded using a Cardio Perfect PC-Based resting ECG system, and bilateral carotid duplex ultrasound; respiratory system examination: chest X-ray and obstructive spirometry; urinary system examination: B-ultrasonography and blood sample and urine assessments; female-specific characteristics: ultrasound, pelvic and gynecologic examinations and the Pap smear. All results are reviewed by two independent experts.

### Follow-up data collection and data management

**Fig. 2:**
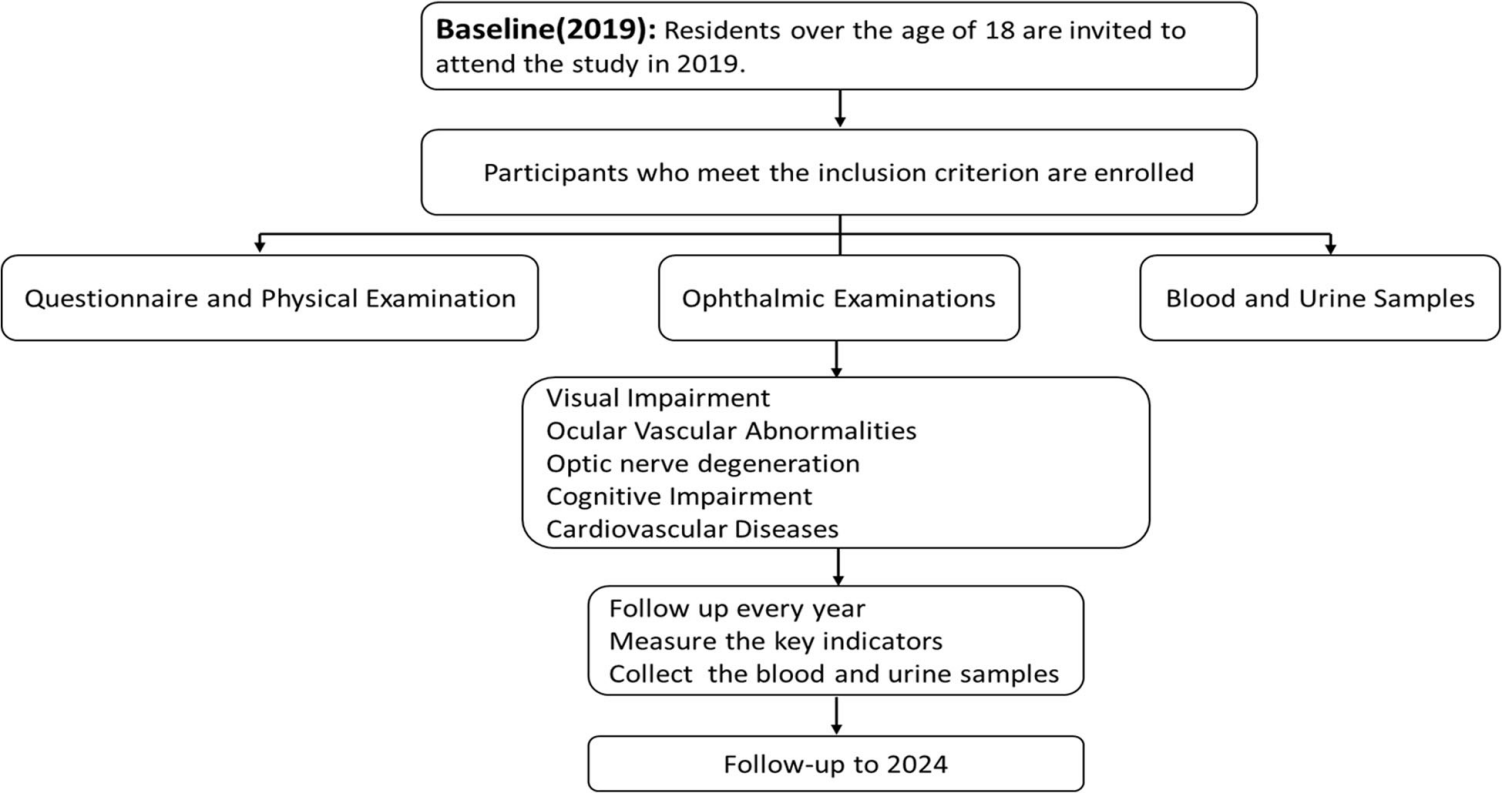
Flow chart of the study

The follow-up period will be 5 years until December 31, 2024. All examinations will be performed by trained researchers or physicians designated by the hospital. Questionnaire, physical examination, ophthalmic examination and collection of biological samples will be conducted annually. Other examinations, such as intraocular pressure and visual field testing, will be tested in subsequent follow-ups. We record the occurrence and progression of visual impairment, collect cardio-cerebrovascular events, and assess neurological alternation such as cognitive impairment. For a detailed flow chart, see Fig. 2.

### Outcome measures

The primary outcome is visual impairment. The level of visual impairment will be assessed by the visual acuity according to the WHO categories (1973) of low vision and blindness and some recent studies [25–28]. Visual impairment can be caused by diverse factors such as opacities of the refractive medium, amblyopia and, particularly, ocular vascular abnormality and optic nerve degeneration. The secondary outcomes include ocular vascular abnormality, optic nerve degeneration, CVD and cognitive impairment. We evaluated ocular vascular abnormality and optic nerve degeneration via comprehensive ocular assessment, particularly digital fundus photography and OCT. Ocular vascular abnormality refers to a series of pathological changes caused by diverse disorders. Additionally, optic nerve degeneration mainly presented as retinal specific layer alternation such as a nerve fiber layer defect (NFLD). All the images and data in this study are processed by two different experienced physicians; when a consensus on diagnosis cannot be reached, a third senior expert will determine the final diagnosis. CVD is diagnosed by physicians including a cardio-cerebrovascular specialist according to the International Classification of Diseases. It mainly includes nonfatal myocardial infarction, nonfatal heart failure, vascular-related operations and surgery, stroke (ischemic stroke, intracerebral hemorrhage, and subarachnoid hemorrhage), systemic embolism events, and cardiovascular death. Additionally, cognitive impairment will be assessed according to MMSE and MoCA.

### Statistical analysis

Continuous variables are expressed as mean ± standard deviation (SD) or median. Categorical variables are expressed as numbers with proportions. Student’s t test or the Wilcoxon rank-sum test is used to compare the difference in the variables of interest between groups. The Chi-squared test is used to evaluate categorical variables. Multivariable analysis is used to evaluate associations between the parameters. The relevant statistical models are used to study the relationships between the variables of interest. Longitudinal analysis is performed to assess the relationship of annual risk factor changes with the outcomes of interest. If necessary, multiple imputation is considered as a replacement for missing values. Statistical analysis is performed using SAS software (version 9.4; SAS Institute, Cary, NC, USA). The level of statistical significance is set at P < 0.05 (two-tailed).

In this report, t-test and Chi-squared tests are used to compare the baseline characteristics between subjects grouped by sex and paired t-test for ocular characteristics between the right and left eyes.

## Results

In total, 4,476 participants from the Jidong communities were initially screened and 1,099 participants among them were excluded due to missing blood samples or an uncompleted questionnaire (119 missing blood samples only, 867 have an uncompleted questionnaire, 113 both). Finally, 3,377 participants aged 45.0 ± 12.5 years were enrolled between August 2019 and January 2020. The baseline characteristics of the included participants are summarized in Table 1. Overall, 53.6% of these participants were women. Most (67.6%) had completed college or higher, and 89.4% had a moderate income between ¥3000 and ¥5000 per month. Among the participants, 18.8% were current smokers and 13.6% were current drinkers. Overall, hypertension occurred in 825 (25.0%), diabetes in 258 (7.7%), hyperglycemia in 474 (14.2%), and CVD history in 100 (3.0%) participants.

**Table 1:** Baseline characteristics of eligible participants in the study

Characteristics	Total	Male	Female	*P* value
n	3377	1568	1809	
Age (SD), years	45.0 (12.5)	45.1 (12.5)	44.9 (12.6)	0.61
Education level, n (%)				< 0.001
Illiteracy / Primary School	108 (3.2)	30 (1.9)	78 (4.3)	
Middle School	978 (29.2)	405 (26.0)	573 (31.9)	
College / University	2268 (67.6)	1122 (72.1)	1146 (63.8)	
Income, n (%)				< 0.001
< ¥3,000	284 (8.5)	91 (5.8)	193 (10.8)	
¥3,000–5,000	2999 (89.4)	1433 (92.0)	1566 (87.2)	
> ¥5,000	70 (2.1)	34 (2.2)	36 (2.0)	
Current Smoking, n (%)	631 (18.8)	615 (39.3)	16 (0.9)	< 0.001
Current Drinking, n (%)	456 (13.6)	447 (28.7)	9 (0.5)	< 0.001
Hypertension, n (%)	825 (25.0)	531 (34.3)	294 (16.8)	< 0.001
Diabetes, n (%)	258 (7.7)	163 (10.5)	95 (5.3)	< 0.001
Hyperglycemia, n (%)	474 (14.2)	292 (18.8)	182 (10.2)	< 0.001
BMI (kg/m^2^)	24.5 ± 3.5	25.6 ± 3.2	23.4 ± 3.3	< 0.001
Fasting Glucose (mmol/L)	5.6 ± 1.4	5.8 ± 1.6	5.4 ± 1.2	< 0.001
Total Cholesterol (mmol/L)	5.0 ± 1.0	5.0 ± 1.0	5.0 ± 1.0	0.781
Triglycerides (mmol/L)	1.8 ± 1.4	2.1 ± 1.6	1.5 ± 1.1	< 0.001
MMSE score	28.3 ± 2.8	28.3 ± 2.7	28.3 ± 2.8	0.96
MoCA score	26.9 ± 3.0	26.9 ± 3.0	26.9 ± 2.1	0.997
CVD history, n (%)	100 (3.0)	50 (3.2)	50 (2.8)	0.532

*BMI* = body mass index, *MMSE* = mini mental status examination scale, *MoCA* = Montreal cognitive assessment, *CVD* = cardiovascular disease

Ocular characteristics of the participants are presented in Table 2. In this report, the right eye characteristics were stated only. For the right eye, the mean spherical equivalent refractive error was −1.9 ± 3.1 diopters, with a mean AL of 24.2 ± 1.4 mm. The mean best-corrected visual acuity (BCVA) was 0.1 logMAR in participants. The average OCT signal index was 8.2 ± 1.2. The Ganglion Cell Complex (GCC) thickness was 103.3 ± 7.9 µm for the entire measured area (0- to 3-mm diameter circle in the macula), while the whole macular thickness was 312.6 ± 13.8 µm. The mean vessel densities were 46.4% and 50.8% for the superficial and deep vascular complexes, respectively. The mean area and perimeter of foveal avascular zone was 0.3 mm^2^ and 2.3 mm, respectively.

**Table 2:** Ocular characteristics of eligible participants in the study

Characteristics	OD	OS	Absolute Difference (95%CI)
BCVA (logMAR)	0.1 ± 0.1	0.1 ± 0.1	0.0 (0.0 – 0.0)
SE (D)	-1.9 ± 3.1	-1.8 ± 2.9	-0.1 (-0.2 – 0.0)
AL (mm)	24.2 ± 1.4	24.2 ± 1.4	0.0 (0.0 – 0.1)
Signal Index	8.2 ± 1.2	8.2 ± 1.3	0.0 (0.0 – 0.0)
Whole GCC thickness (μm)	103.3 ± 7.9	103.2 ± 7.8	0.1 (-0.1 – 0.3)
Whole Macular thickness (μm)	312.6 ± 13.8	312.7 ± 13.8	0.0 (-0.5 – 0.4)
Whole SCP Vessel Density (%)	46.4 ± 3.2	46.2 ± 3.4	0.0 (-0.2 – 0.1)
Whole DCP Vessel Density (%)	50.8 ± 3.3	51.0 ± 3.6	-0.3 (-0.4 – -0.1)
Foveal Avascular Zone Area (mm^2^)	0.3 ± 0.2	0.3 ± 0.3	0.0 (0.0 – 0.0)
Foveal Avascular Zone Perimeter (mm)	2.3 ± 0.6	2.3 ± 0.6	0.0 (0.0 – 0.0)

*BCVA* = best-collect visual acuity, *SE* = spherical equivalent, *AL* = axial length, *GCC* = ganglion cell complex, *SCP* = superficial capillary plexus, *DCP* = deep capillary plexus

## Discussion

The JECS enrolled 3,377 participants from August 2019 to January 2020. The participant baseline characteristics and ocular characteristics were reported. Compared with several existing population-based cohorts, [29–37] the JECS is a unique and high-quality study focusing on ocular assessment along with comprehensive etiological evaluation, imaging and biomarker collection, providing the opportunity to identify risk factors, imaging and biomarkers of visual impairment (either ocular vascular anomalies or optic nerve degeneration) and to evaluate their associations with CVD and neurological diseases. Data from the JECS will provide evidence to formulate recommendations for optimal practice not only in visual impairment precaution but also in CVD and neurological diseases prevention (eye as a window) in subsequent phases. The findings from the JECS will be updated to improve the clinical performance measures. The JECS would formulate the collaboration in clinical practice for ophthalmologists and cardiologists or neurologists. Vision health and general health of a person will be considered by ophthalmologists. Meanwhile, cardiologists and neurologists, will identify more alternatives for diagnosing CVD and neurological diseases from the eye.

The JECS has several strengths. First, a series of detailed ocular measurements is performed on participants, such as OCT angiography. In most cases, the small retina vessels and capillaries are first affected and pathologically changed due to CVD risk factors and various metabolic disorders [38, 39]. OCT angiography enables the visualization and quantitative analysis of retinal capillaries. Second, several relatively detailed cardio-cerebrovascular and neurological screening tests make it feasible to identify the association between vision impairment and CVD or cognitive impairment. The retinal vasculature shares many features with cardiovascular or cerebral vessels and is often exposed to the same environmental and intrinsic influences [40]. Therefore, the eye is a window to the heart or brain given its easily accessible vasculature. Additionally, certain unique vascular features in the retina are essential. Third, lifestyles of the participants in the JECS are collected through face-to-face interviews and follow-up and is performed annually. Finally, the longitudinal study will provide evidence to reveal the occurrence and development of ocular vascular abnormalities and optic nerve degeneration not only in ocular disease but also in cardio-cerebrovascular disease and cognitive impairment after several years of follow-up. The JECS contributes to the extensive evaluation of objective health conditions as well as environmental factors.

Our study also has several potential limitations. First, a selection bias may exist because the study was performed at a center where the economy is relatively developed and the population density is high. Moreover, some participants are recruited among the employees and retirees of the Jidong Oil-Field of Chinese National Petroleum Company who may not fully represent the entire Chinese population. Second, some of the data may be missing or subjects may be lost to follow-up due to the large number of examination items in this study. Considering this, we intensified the supervision to ensure the integrity of all the examination data. Additionally, the participants who would be followed up were notified in advance by telephone calls combined with text message reminders to reduce the rate of follow-up loss.

## Conclusions

The JECS is a community-based, prospective, long-term follow-up observational study designed to identify the etiology, imaging and biomarkers for visual impairment and to evaluate their associations with risks of CVD and neurological diseases. The findings from the JECS will provide evidence for the risk factors of vascular abnormalities or neurodegeneration and further promote the establishment of early warning models of disease.

## Data Availability

The datasets used and/or analyzed during the current study are available from the corresponding author upon reasonable request. The data that support the findings of this study are also available from Ruike Donghua Translational Medical Research Center.

## Abbreviations

JECS: Jidong Eye Cohort Study
OCT: Optical coherence tomography
CVD: Cardiovascular diseases
MMSE: Mini Mental Status Examination Scale
MoCA: Montreal Cognitive Assessment
BMI: Body mass index
ACC: American College of Cardiology
AHA: American Heart Association
HBA1c: Hemoglobin A1c
TC: Total cholesterol
TG: Total triglycerides
HDL-C: High-density lipoprotein
LDL-C: Low-density lipoprotein
Scr: Serum creatinine
UA: Uric acid
VA: Visual acuity
CCT: Central corneal thickness
AD: Aqueous depth
LT: Lens thickness
AL: Axial length
LOCS III: Lens Opacities Classification System
RCP: Retinal complex perfusion
OCTA: Optical coherence tomography angiography
ECG: Electrocardiogram
NFLD: Nerve fiber layer defect
SD: Standard deviation
BCVA: Best-corrected visual acuity
GCC: Ganglion cell complex
